# Red cells distribution width after cardiac arrest

**DOI:** 10.1186/2197-425X-3-S1-A202

**Published:** 2015-10-01

**Authors:** V Fontana, P Villois, C Righy Shinotsuka, L Nobile, J-L Vincent, J Creteur, FS Taccone

**Affiliations:** Erasme University Hospital, Bruxelles, Belgium

## Intr

Red cell distribution width (RDW) is a quantitative measure of the variability in size of erythrocytes and it is used for the differential diagnosis of anemia. Moreover, in critically ill patients, high RDW values have been associated with increased hospital mortality. No data on the impact of RDW on the outcome of patients resuscitated from cardiac arrest (CA) are available.

## Objectives

To investigate whether RDW values are associated with outcome after CA.

## Methods

Retrospective analysis of an institutional database including all adult comatose patients admitted to the Intensive Care Unit (ICU) after CA from January 2007 to December 2014. Inclusion criteria were as follows: age ≥18, non-traumatic arrest and survival ≥ 24 hours after admission. We collected demographics and CA related data. We also collected daily routine blood analyses; RDW (normal values: 10.9-13.4%), was obtained from the day of admission to day 3. We recorded ICU mortality and long-term neurological outcome; a CPC score of 3-5 at 3-months was used to define poor neurological outcome (PNO).

## Results

Of the 404 eligible patients, 14 were excluded because of lack of blood analyses and 390 were eventually included. Median age was 62 [52-75] years and 275 (70%) were male. Median time to ROSC was 15 [8-25] minutes and 241 (62%) patients had a non-shockable initial rhythm. ICU mortality was 56% (n = 220) and 3-month PNO was observed in 251 (64%) patients. Median RDW on the day of admission was 14 [13.0-15.2]% and remained stable over the observation period. RDW on admission was within normal ranges in 145 (37%) patients. We found a significantly higher RDW on admission in non-survivors than survivors (14.2 [13.2-15.5]% vs. 13.7 [12.6-14.9]%; p = 0.0007) as well as in patients with PNO when compared to those with favourable outcome (14.2 [13.1-15.5]% vs. 13.6 [12.6-14.8]%; p = 0.001). ICU mortality increased with increasing RDW quartiles (from 42% for RDW ≤12.9% [Quartile I] to 65% for RDW ≥15.6% [Quartile IV]; p = 0.001) as well as PNO (from 51% to 72% for the same RDW ranges; p = 0.003).

## Conclusions

RDW values are higher in non-survivors and patients with poor neurological outcome after CA. Patients with the highest RDW values will have a higher mortality rate and worse neurological recovery.

